# Tumor Necrosis Factor-**α** and Interleukin-6: Potential Interorgan Inflammatory Mediators Contributing to Destructive Periodontal Disease in Obesity or Metabolic Syndrome

**DOI:** 10.1155/2013/728987

**Published:** 2013-08-28

**Authors:** Roozbeh Khosravi, Khady Ka, Ting Huang, Saeed Khalili, Bich Hong Nguyen, Belinda Nicolau, Simon D. Tran

**Affiliations:** ^1^Faculty of Dentistry, McGill University, 3640 University Street, M43, Montreal, Quebec, Canada H3A 0C7; ^2^University of Toronto, Toronto, Canada; ^3^Department of Pediatrics, CHU Sainte-Justine, University of Montreal, 3175 Cote-Ste-Catherine, Montreal, Quebec, Canada H3T 1C5

## Abstract

Obesity has become a worldwide health burden in the last two decades. Obesity has been associated with increased comorbidities, such as coronary artery disease, diabetes, and destructive periodontal disease. Obesity is also part of a group of risk factors occurring together in an individual, which is referred to as metabolic syndrome. Clinical studies have shown higher risk for destructive periodontal disease in obesity and metabolic syndrome. However, the role of obesity and metabolic syndrome in the onset and development of destructive periodontal disease has not yet been fully understood. In this review, we discuss a working model, which focuses on interorgan inflammation as a common etiological factor for destructive periodontal disease associated with obesity and metabolic syndrome. Specifically, we suggest that elevated levels of tumor necrosis factor-**α** (TNF-**α**) or interleukin 6 (IL-6)—both adipokines and known risk factors for destructive periodontal disease—in obesity and metabolic syndrome contribute to the onset and development of destructive periodontal disease. The connections between destructive periodontal disease and systemic conditions, such as obesity or metabolic syndrome, are complex and potentially multidirectional. This review largely focuses on TNF-**α** and IL-6, inflammatory mediators, as potential common risk factors and does not exclude other biological mechanisms.

## 1. Periodontal Diseases

Periodontal diseases are inflammatory diseases affecting the surrounding and supporting tissues of teeth—the periodontium. Gingivitis and destructive periodontal disease (periodontitis) are the two most common forms of periodontal diseases. Gingivitis is an inflammatory reaction often induced by the pathogens residing in dental plaque (biofilm), which forms on the adjacent tooth surfaces [1]. Destructive periodontal disease results in an apical loss of epithelial attachment along with the periodontal soft and hard tissues [2]. Unlike gingivitis, which is cured following the removal of local etiological factors, destructive periodontal disease is irreversible. Destructive periodontal disease is mediated by various intrinsic and acquired factors; two individuals with similar microbiological profile could show different susceptibility to periodontal diseases [3]. Several case-control and cohort studies have reported the contribution of systemic conditions and diseases in the onset and exacerbation of destruction periodontal disease. Preterm birth [4], cardiovascular diseases [5], and diabetes [6] are examples of these conditions. In addition, a growing body of evidence during the last decade suggests obesity as a risk factor for destructive periodontal disease [7, 8]. Metabolic syndrome has also been shown to be positively associated with destructive periodontal disease [9–22]. Although the majority of studies on destructive periodontal disease in individuals with obesity or metabolic syndrome concentrated on adults, some studies reported on evidence proposing that this potential link in children and adolescence exists [23]. In this paper, we review the evidence suggesting that destructive periodontal disease is linked to obesity and metabolic syndrome, as an example of interorgan crosstalk under inflammatory conditions. Additionally, we discuss a working biological model on the onset of destructive periodontal disease in individuals with obesity or metabolic syndrome based on elevated levels of tumor necrosis factor-*α* (TNF-*α*) and interleukin 6 (IL-6) in these conditions.

## 2. Obesity

Obesity is a multifactorial condition with a wide range of etiological factors including genetic, biological, social, and behavioral factors, all of which likely interact to ultimately lead to a chronic imbalance between energy intake and energy expenditure. This imbalance could cause excessive fat accumulation and result in adverse health consequences. Obesity has reached epidemic proportion worldwide, largely because of increased consumption of high caloric diet and a sedentary lifestyle. According to the World Health Organization [24], approximately 2.3 billion adults will be overweight and more than 700 million will be obese by 2015. This phenomenon affects particularly developed countries. Over the past two decades, the overall obesity rates have reached 24.1% and 34.4% in Canada and the United States, respectively [25].

Based on current Health Canada guidelines, a body mass index (BMI, kg/m^2^) of 25–30 and over 30 are considered overweight and obese, respectively [26]. Obesity is categorized into 3 classes according to the increased health risks associated with increasing BMI levels: class I (BMI 30–34.9), class II (BMI 35–39.9), and class III (BMI ≥ 40) [27]. Pediatric obesity has also become a public health concern since it is more common for children to experience the negative health consequences of obesity, which used to be only seen in adulthood. In 2010, more than 40 million children under the age of five were estimated to be overweight worldwide [28]. In 2004, 26% of Canadian children and adolescents aged 2 to 17 years were overweight or obese [29]. Obesity has been associated with a wide spectrum of comorbidities, such as coronary artery disease, strokes, diabetes, arthritis, reproductive dysfunctions, and various cancers [30].

## 3. Metabolic Syndrome

Metabolic syndrome is a cluster of risk factors (abdominal obesity, diabetes, high cholesterol, high blood pressure, and raised fasting glucose) that increases the chance of developing type 2 diabetes and cardiovascular diseases [31]. Metabolic syndrome is also known as syndrome X, insulin resistance syndrome, dysmetabolic syndrome X, Reaven's syndrome, multiple metabolic syndrome, and metabolic cardiovascular syndrome [18, 32–34]. Several definitions for metabolic syndrome in adult populations have been proposed by different organizations including the World Health Organization (WHO), the International Diabetes Federation (IDF), the American Heart Association/National Heart, Lung, and Blood Institute (AHA/NHLBI), the National Cholesterol Education Program Adult Treatment Panel III (NCEP-ATP III), and the European Group for the Study of Insulin Resistance (EGIR) [15]. Although these diagnostic criteria are similar, there is variability with the different cut-off values set for each criterion. The unified criteria to diagnose metabolic syndrome include at least 3 of the following: abdominal obesity, high level of plasma triglycerides, low level of high-density lipoprotein (HDL) cholesterol, high blood pressure, and impaired fasting glucose or insulin resistance [35].

The reported prevalence of metabolic syndrome varies depending on the diagnostic criteria opted by different studies, nonetheless the prevalence of metabolic syndrome has increased at epidemic rates for the past decade. Findings from the National Health and Nutrition Examination Survey (NHANES) 2003–2006 showed that 34% of Americans, 20 years and older, met the criteria to be diagnosed with metabolic syndrome based on the NCEP-ATP III definition [36]. Canadian Health Measures Survey revealed that about one in five Canadian adults (20% of the population) had metabolic syndrome [37]. No standard diagnostic criteria for metabolic syndrome in children and adolescents have been developed, which makes studying metabolic syndrome in children challenging. Nonetheless, the Third National Health and Nutrition Examination Survey (NHANES III, 1988–1994) reported on a 4.2% prevalence of metabolic syndrome among 12- to 19-year-old American adolescents (approximately 1 million adolescents) [38]. In addition, nearly 30% of overweight adolescents met the criteria of metabolic syndrome according the definition proposed by the NCEP-ATPIII [38]. De Ferranti and colleagues reported an increase of 38% in the prevalence of metabolic syndrome in adolescents of the same age group between 1988 and 2000 using the same definition [39].

## 4. Periodontal Diseases in Obesity and Metabolic Syndrome

**Figure 1:**
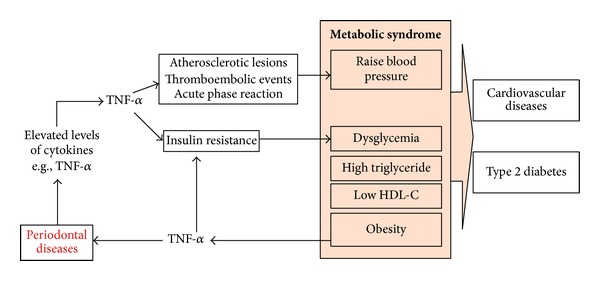
A biological working model on the onset of destructive periodontal disease in obesity and metabolic syndrome adapted from Nishimura et al., 2003 [8]. This scheme represents a potential working model in which the systemic elevated levels of TNF-*α* in obesity and metabolic syndrome potentially contribute to the onset and development of destructive periodontal disease. Specifically, the elevated levels of systemic inflammatory mediators, such as TNF-*α* or IL-6, in obesity or metabolic syndrome enhance the host response to periodontal pathogens hence increase the chance to develop destructive periodontal disease. In destructive periodontal disease, periodontal pathogens induce inflammation prompting the destruction of connective tissues and bone in the periodontium. The connections between destructive periodontal disease and systemic conditions, such as obesity, are complex and often multidirectional; the working model presented here is a simplified picture of these connections.

Periodontal diseases, a chronic inflammation by its nature, have been linked to many systemic conditions. The findings from meta-analyses suggest that cardiovascular diseases [40–42] and obesity [43–45] increase the chance of destructive periodontal disease by 20% and 35%, respectively. Moreover, studies on diabetic patients indicate that type 2 diabetes is a risk factor for advanced form of destruction periodontal disease [46]. Metabolic syndrome is also positively correlated with destructive periodontal disease [9–22]. A recent meta-analysis reported that the odds of destructive periodontal disease to occur was 1.71 to 2.09 higher in individuals with metabolic syndrome compared to those without this syndrome [47]. Despite all these evidence indicating the higher risk of destructive periodontal disease in obesity and metabolic syndrome, the underlying biological mechanism(s) is yet to be fully understood. Studying the common etiological factors in obesity, metabolic syndrome, and destructive periodontal disease would be a potential approach to delineate biological mechanisms explaining the higher risk of destructive periodontal disease under these conditions. Inflammation is indeed one of the common factors in the pathogenesis of destructive periodontal disease, obesity, and metabolic syndrome.  Figure 1 represents a simplified working model explaining how inflammation connects these diseases. Indeed, this working model does not exclude other potential biological mechanisms.

In this review we focus on a potential interorgan inflammatory mechanism explaining the higher prevalence of destructive periodontal disease in obesity and metabolic syndrome. Higher risk of destructive periodontal disease associated with obesity and/or metabolic syndrome is most likely bidirectional, and here we only concentrate on how obesity or metabolic syndrome induces or enhances destructive periodontal disease. 

Tumor necrosis factor-*α* (TNF-*α*) is the best candidate connecting higher destructive periodontal disease in obesity or metabolic syndrome. TNF-*α* levels are systemically elevated in both obesity and metabolic syndrome [48]. Studies on human and animal models in the 1990s indicated that adipocytes secrete TNF-*α*, and hence the excess of fat in obesity leads to a systemic chronic inflammation [49, 50]. TNF-*α* was also reported to induce insulin resistance in both diabetes and obesity [51]. It is known that other cytokines from adipose tissues also contribute to the chronic systemic inflammation in obesity [48]. 

TNF-*α* is one of the key periodontal pathogens-induced early inflammatory cytokines in destructive periodontal disease [52]. Elevated levels of TNF-*α* are a well-known risk factor for destruction periodontal disease [52]. Increased levels of TNF-*α* contribute to the onset of destructive periodontal disease via several mechanisms. Examples of these mechanisms are (i) TNF-*α* prompts the destruction of alveolar bone by stimulating the formation of bone-resorbing cells (osteoclasts) [53]; (ii) TNF-*α*, as one of the early promoters of host response to periodontal bacterial pathogens, regulates matrix metalloproteinases (MMPs), which are capable of degrading the connective tissues. Interestingly, studies on the immune response to periodontal pathogens showed that TNF-*α* enhanced the immune response to these pathogens [54]. Previous studies on human subjects reported on the elevated levels of TNF-*α* in gingival crevicular fluid (GCF) of obese individuals. The authors reported on a 0.74 pg increase in GCF TNF-*α* with an increase of one BMI unit. 32 obese subjects aged between 13 and 24 years was recruited in this study [55]. In a larger scale study on the third National Health and Nutrition Examination Survey (NHANES II) dataset, Genco and colleagues showed that serum TNF-*α* levels were not correlated with the severity of destructive periodontal disease in BMI over 30 kg/m, and proposed the notion that TNF-*α* mainly contributes to the initial stage of destructive periodontal disease development [56]. In addition, our group has been investigated the potential development of destructive periodontal disease in children at risk of obesity and metabolic syndrome as they age. We are studying the Oral Health component within a cohort of 600 children in Quebec (the Quebec Adipose and Lifestyle Investigation in Youth; QUALITY cohort), which aims to investigate the natural history of obesity and its vascular and metabolic consequences [57]. Our results from the baseline visit of the QUALITY cohort so far indicate that obese boys, but not girls, have 37% higher levels of TNF-*α*, a risk factor for destructive periodontal disease, in their gingival crevicular fluid (GCF) compared to nonobese boys [58]. We also found that metabolic syndrome in boys was associated with 49 percent increase in the levels of TNF-*α* in gingiva crevicular fluid (Ka et al.; unpublished data).

The second candidate explaining a mechanism for the higher prevalence of destructive periodontal diseases in obesity or metabolic syndrome is interleukin 6 (IL-6). IL-6 is a multifunctional cytokine produced by variety of cells including macrophages, neutrophils, and endothelial cells (reviewed in [59]). The double edge effects (i.e., pro- and anti-inflammatory) of IL-6 create a complexity in investigating its roles in normal or diseased conditions [59]. IL-6 systemic and GCF levels increase in destructive periodontal diseases (reviewed in [60]). Additionally, controversial studies suggested an increase or no change in serum IL-6 levels in obesity and metabolic syndrome (reviewed in [61]). Unfortunately, to the best of our knowledge, no evidence from animal or human studies supports the hypothetical model in which obesity or metabolic syndrome-induced IL-6 increases the risk of destructive periodontal disease.

Taken together, these evidence support the notion that, in obesity and metabolic syndrome, the elevated levels of TNF-*α*, and possibly IL-6, may increase the chance of destructive periodontal diseases development, which could be directly through mechanisms discussed earlier and also indirectly by enhancing the bacterial-induced host immune response in obesity and metabolic syndrome.

## 5. Conclusion

The obesity epidemic in North America and worldwide is an alarming public health issue. The prevalence of childhood obesity and overweight is rising rapidly in Canada [62, 63]. The total direct costs attributable to overweight and obesity in Canada was $6.0 billion in 2006, representing 4.1% of the total health expenditures in the country [64]. Similarly, data from American adults showed an increase in the prevalence of metabolic syndrome from 24 to 27% between 1988–1994 and 1999–2000 [65]. Similar trends were seen in American adolescents particularly in overweight and obese subjects, for whom the prevalence of metabolic syndrome was 32.1% [32]. Moreover, higher health care cost with increased number of metabolic syndrome components has been reported [66–68]. The annual health care cost associated for an individual with 1 component was estimated to be $5564 compared to $12,287 for someone with 4 components [68].

Understanding the multidirectional and dynamic links among obesity, metabolic syndrome, and destructive periodontal disease can improve current preventive and therapeutic modalities for these conditions. For example, one could screen the GCF TNF-*α* levels of obese individuals to identify a subgroup of obese subjects, who are more susceptible to develop destructive periodontal disease.
